# The costs of inequality: whole-population modelling study of lifetime inpatient hospital costs in the English National Health Service by level of neighbourhood deprivation

**DOI:** 10.1136/jech-2016-207447

**Published:** 2016-05-17

**Authors:** Miqdad Asaria, Tim Doran, Richard Cookson

**Affiliations:** 1 Centre for Health Economics, University of York, York, UK; 2 Department of Health Sciences, University of York, York, UK

**Keywords:** ECONOMICS, Health inequalities, INEQUALITIES, NHS, SOCIO-ECONOMIC

## Abstract

**Background:**

There are substantial socioeconomic inequalities in both life expectancy and healthcare use in England. In this study, we describe how these two sets of inequalities interact by estimating the social gradient in hospital costs across the life course.

**Methods:**

Hospital episode statistics, population and index of multiple deprivation data were combined at lower-layer super output area level to estimate inpatient hospital costs for 2011/2012 by age, sex and deprivation quintile. Survival curves were estimated for each of the deprivation groups and used to estimate expected annual costs and cumulative lifetime costs.

**Results:**

A steep social gradient was observed in overall inpatient hospital admissions, with rates ranging from 31 298/100 000 population in the most affluent fifth of areas to 43 385 in the most deprived fifth. This gradient was steeper for emergency than for elective admissions. The total cost associated with this inequality in 2011/2012 was £4.8 billion. A social gradient was also observed in the modelled lifetime costs where the lower life expectancy was not sufficient to outweigh the higher average costs in the more deprived populations. Lifetime costs for women were 14% greater than for men, due to higher costs in the reproductive years and greater life expectancy.

**Conclusions:**

Socioeconomic inequalities result in increased morbidity and decreased life expectancy. Interventions to reduce inequality and improve health in more deprived neighbourhoods have the potential to save money for health systems not only within years but across peoples’ entire lifetimes, despite increased costs due to longer life expectancies.

## Introduction

Healthcare systems in most high-income countries aspire to provide equitable care, adopting the principle of equal access to services for equal need,^1^ even when this is difficult to define and implement in practice.^2^ Some, such as the National Health Service (NHS) in England go further, and aim for equal use of healthcare or even equal outcomes.^3^ However, health status is powerfully influenced by socioeconomic factors, with lower income associated with greater healthcare needs.^4^ So for a system to be equitable it must de-couple use of healthcare services from individual income and contributions towards system costs. This is usually achieved through social insurance schemes, or—as in the case of the English NHS—by funding system costs through progressive income taxation. Through the use of such funding arrangements, healthier people subsidise care for those who fall ill, and more affluent sections of society subsidise the more deprived.

There is a widespread assumption that over the life course such systems disproportionately favour people lower down the socioeconomic scale, in terms of the imbalance between their contribution to the costs of health services and their use of those services.^5^ Lower socioeconomic status is associated with lower incomes, and therefore, smaller income tax and social insurance contributions, but also with greater healthcare need, in particular, the earlier development of multiple chronic morbidities.^6^ ^7^ However, evidence on actual use of services is more nuanced. More deprived populations tend to make greater use of unplanned (emergency) services than affluent populations, and are slightly more likely to visit the GP,^8^ but are less likely to visit a medical specialist or to use many types of planned and preventative services.^9^

Most studies, to date, on the costs and use of healthcare services by different socioeconomic groups have been cross-sectional. This is an important limitation, because morbidity and mortality may have opposing impacts on lifetime healthcare costs—greater morbidity will tend to increase lifetime costs, whereas dying younger will tend to reduce them. After early childhood, average current-year healthcare costs for individuals increase throughout life, rising dramatically from the age of 50.^10^ These higher healthcare costs for poorer people in life may be partially offset by a shorter lifespan. Alternatively, given that the rising costs in older age are largely driven by the onset of chronic disease, earlier onset of these diseases in poorer populations may simply shift the healthcare costs to younger age groups.

Consideration of these longitudinal relationships is necessary in order to determine the impact of socioeconomic factors on health system costs. Measuring the size of this impact is important not just to quantify the relative healthcare benefits received by different social groups, but to understand the costs borne by the health service as a consequence of social inequality. In this study, we aimed to measure the costs to the NHS of socioeconomic inequality, by estimating the lifetime inpatient hospital costs of the whole English population by socioeconomic status.

## Methods

### Data

This study focuses on socioeconomic differences in inpatient hospital costs across the life course. Hospital admissions in England are recorded in the Hospital episode statistics (HES) data set used to reimburse hospitals for provided care. This data set contains details on every episode of care, and a new finished consultant episode (FCE) record is created for every new admission, and every time responsibility for the care of a patient passes from one consultant to another. The HES FCE records data about the patient (age, sex, place of residence) and their hospital stay (diagnoses, procedures, length of stay). Using this information the FCE is allocated to a healthcare resource group (HRG), which collates hospital stays that use similar levels of resources—this is the English version of diagnosis related groups used in the USA. Hospitals are reimbursed by the NHS through the payments by results (PbR) system based on the HRG, adjusted for the specifics of the case—for example, a more complicated case with longer than usual length of stay attracts additional reimbursement. Reimbursement is also adjusted for local cost variations (termed ‘market forces factors’). Costs attached to each HRG for each year, and variations for more complex cases, are given in the NHS national reference costs.^11^ Details of how to derive costs from HES data are available in the PbR documentation,^12^ and their use in health economic analysis is discussed in Asaria *et al*.^13^ We use HES inpatient data for 2011/2012 and associated reference costs in this study.

The basic geographical unit of analysis in this study is the lower-layer super output area (LSOA). The country is divided into 32 482 LSOAs each containing, on average, 1500 people (range 1000–3000). Population data for 2011/2012 are taken from Office for National Statistics (ONS) midyear population estimates split by LSOA, sex and age (ages 0–84 in single-year estimates, and then 85+). This data estimates the total resident population, including homeless people and people living in institutions. Mortality data for 2011/2012 are taken from the ONS, split by LSOA, sex and age (ages 0–84 in 5-year age bands, and then 85+). Area deprivation for LSOAs is measured using the index of multiple deprivation (IMD) for 2010. The IMD includes seven domains: (1) income; (2) employment; (3) health deprivation and disability; (4) education skills and training; (5) barriers to housing and services; (6) crime; and (7) living environment. These domains are combined to produce an overall deprivation rank for each LSOA. We grouped LSOAs into deprivation quintiles based on this rank ranging from Q1 (the most deprived fifth of LSOAs) to Q5 (the least deprived fifth of LSOAs).

### Analysis

We grouped HES inpatient data into age, sex and IMD quintile categories. Of the 18 808 903 episodes in our 2011/2012 HES data set, 1 659 295 episodes (8.8%) could not be grouped due to missing data on either age, sex or LSOA of residence, and were dropped from the analysis. We then calculated the total cost for each age, sex and IMD quintile group using the HRGs and the relevant reference costs. Market forces factors adjustments were not made as we are interested in the variation in resource use by deprivation group rather than local cost variations. We then inflated these costs by 8.8% to account for the missing data (we assumed that missing data were equally distributed across all patient groups and HRGs). Finally, we divided by the population in each age, sex and IMD quintile group using ONS population estimates to estimate average costs for each group:
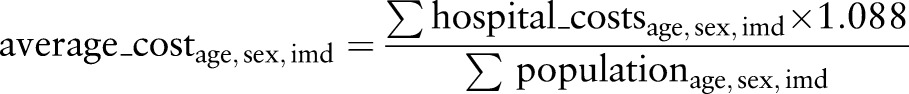


We used these average costs to calculate the total cost associated with inequality in 2011/2012 by comparing the costs as observed in the data with the costs calculated, by assuming that each individual experienced the average costs (split by age and sex) experienced in the least deprived fifth of areas:
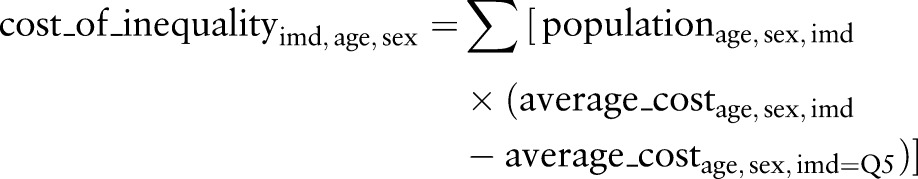
Next we used the mortality data to calculate mortality rates by age, sex and IMD quintile group and used these in turn to calculate survival curves for each group:
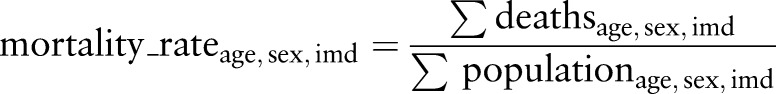


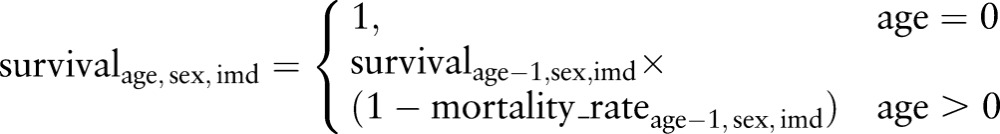


We used these survival curves to calculate expected cost at each age split by sex and IMD quintile group by adjusting the average cost for the probability of an individual from each group being alive to incur that cost. Finally, we summed across these age groups to get an expected lifetime cost for an individual in each sex and IMD quintile group (assuming mortality experience and hospital costs remained constant at 2011/2012 level):
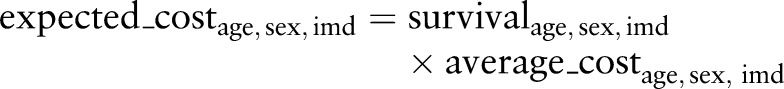


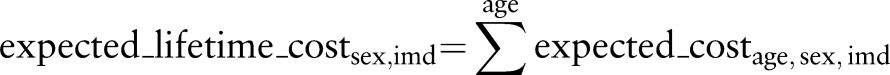


We repeated this analysis for emergency and elective hospitalisations, and also compared rates of outpatient hospital use among the different groups.

The analysis was performed using Oracle 11g and R 3.2.3—the analysis code is available at https://github.com/miqdadasaria/cost-of-inequality

## Results

### Social patterning of hospital episodes

**Figure 1:**
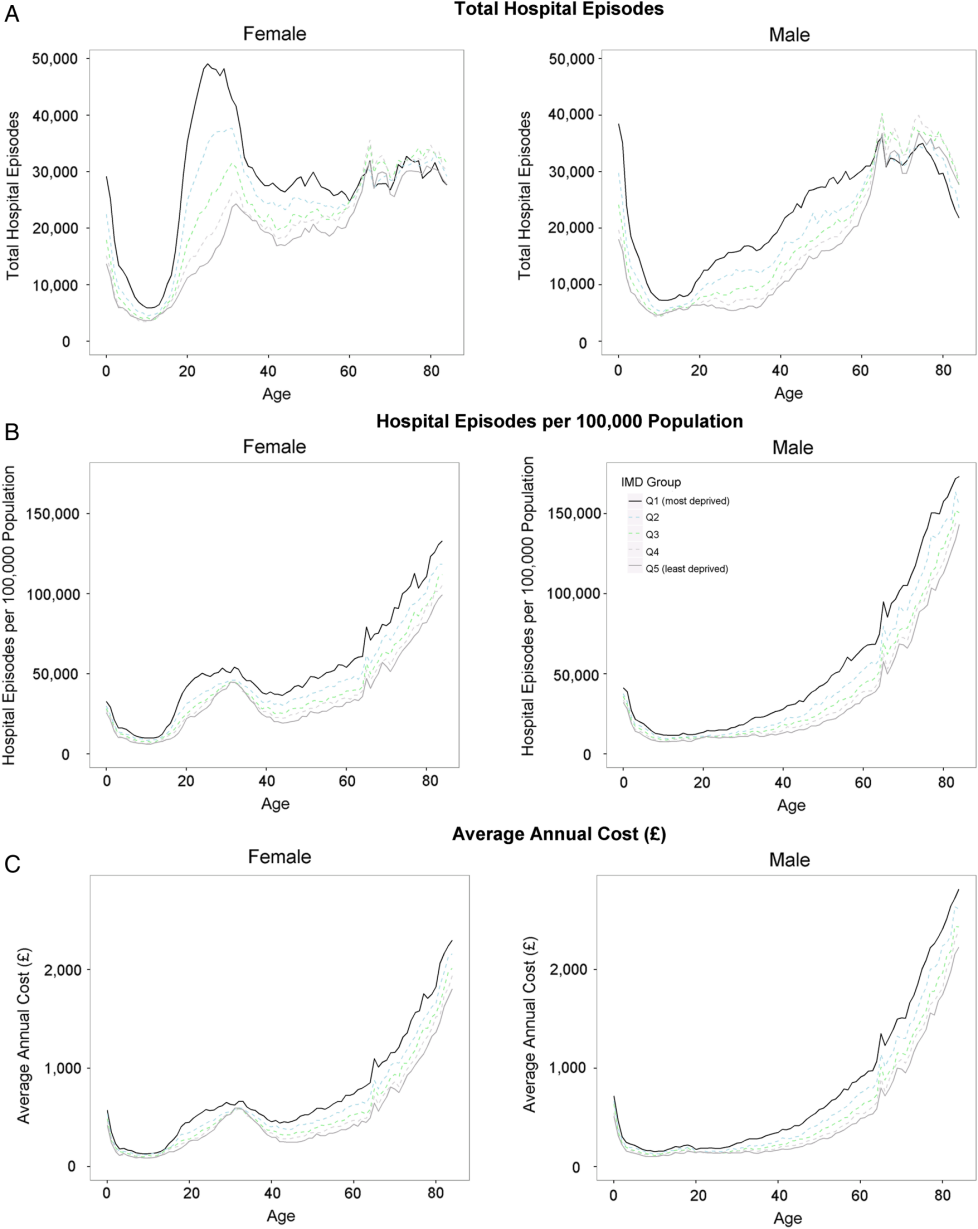
All hospital inpatient admissions split by age, sex and deprivation. Graphs are based on hospital episode statistics for year 2011/2012 and are broken down by sex (female on the left male on the right), deprivation (different line colours) and are plotted against age. (A) Shows the total number of hospital episodes. (B) Shows the hospitalisation rate that is, adjusts for the demographic structure of the population. (C) Translates from hospital episodes to average annual costs due to these hospitalisations.

In 2011/2012, there were 11 477 435 elective episodes and 7 914 736 emergency episodes to hospitals in England (19 392 171 total episodes). Numbers of episodes decreased between the ages of 0 years and 10 years in both sexes, then, for men, increased up to the age of 70 years, before declining in the oldest age groups, and for women, spiked sharply between adolescence and the age of 40 years —reflecting admissions relating to reproduction—before gradually increasing up to the oldest age groups (figure 1A). For ages 0 years–60 years, there was a clear social gradient in both sexes, with episodes increasing with area deprivation. After the age of 60 years, this trend began to reverse until in the over 75 years age group the most deprived areas had the fewest episodes. The greatest gap between social groups occurred in women during the peak reproductive years.

**Figure 2:**
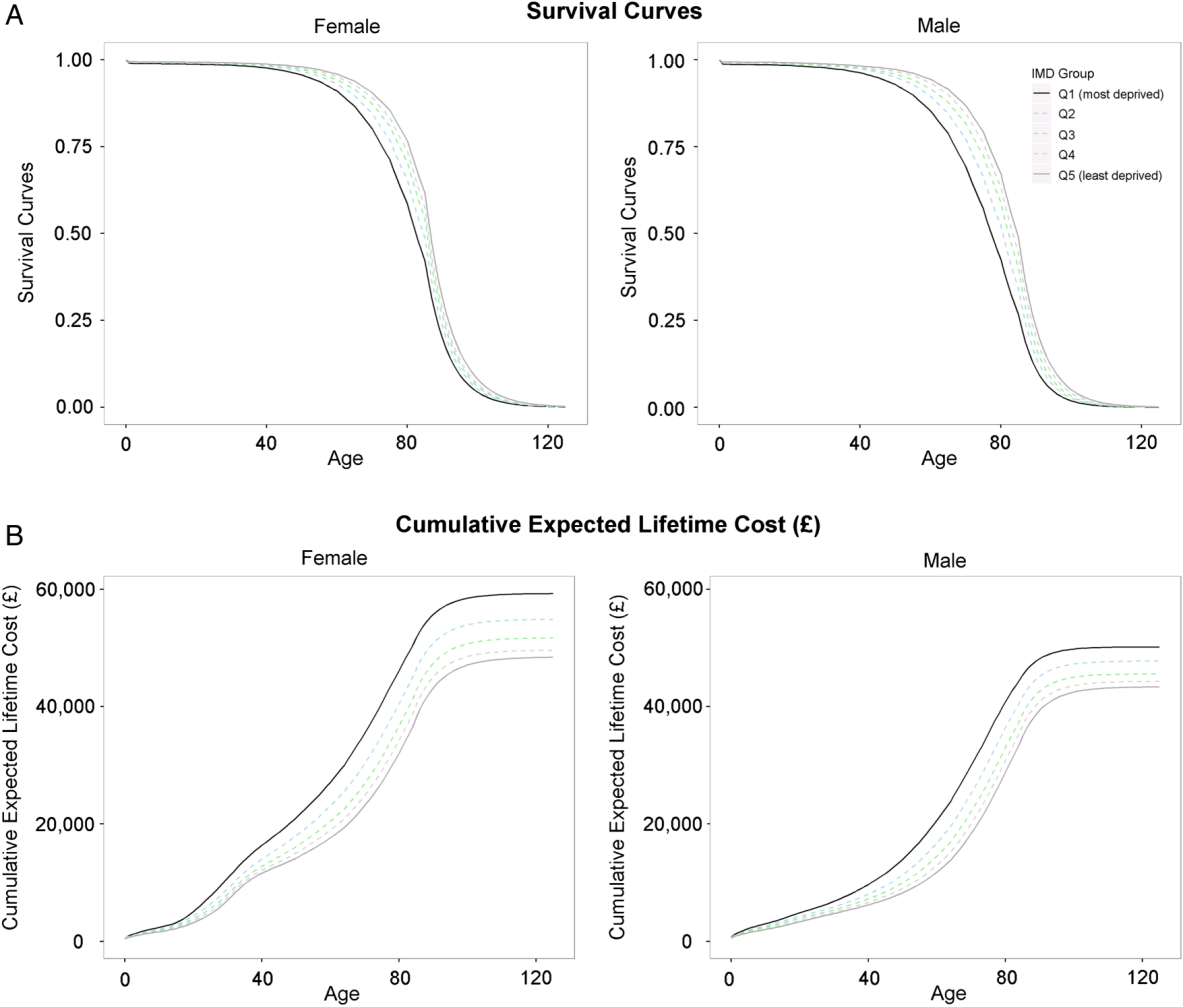
Survival curves and cumulative lifetime costs split by age, sex and deprivation. Graphs are based on mortality data and hospital episode statistics for year 2011/2012, and are broken down by sex (female on the left male on the right), deprivation (different line colours) and are plotted against age. (A) Shows the probability of surviving against age. (B) Shows the cumulative expected hospital costs calculated by adjusting hospital costs by the probability of being alive at any given age and cumulating these adjusted costs over all previous years.

Figure 1B shows the rate of episodes after adjusting for the different demographic structures of population groups. After early childhood, rates of hospital episodes generally increased with age, and were higher in women than in men between the ages of 20 years and 40 years, and higher in men after the age of 70 years. A social gradient was again evident with a higher rate of episodes in more deprived areas, but in the case of episode rates, the gradient persisted across the entire age range. This indicates that the relative fall in the number of episodes for older age groups in more deprived areas was due to a relative decline in population, with fewer people in deprived areas surviving into old age (figure 2A).

The trends for average annual costs per head of population (figure 1C) closely mirrored the patterns for hospital episode rates, suggesting that costs associated with different population groups were primarily driven by volumes of hospital usage rather than differences in types of hospital usage across the life course.

The social gradient in hospital episodes was evident for elective and emergency admissions, but the gaps were greater for emergency admissions (table 1**)**. Compared with residents in the most affluent fifth of areas, residents of the most deprived fifth of areas had a 20% higher rate of elective episodes, a 71% higher rate of emergency episodes, and a 39% higher rate of episodes overall. Detailed age, sex and deprivation breakdowns of the different types of admissions are given in the online supplementary appendix figure A1.

**Table 1:** Number and rate of hospital episodes by admission type

IMD quintile	Elective		Emergency		All	
	Total	Rate*	Total	Rate*	Total	Rate*
Q1 (most deprived)	2 481 014	23 727	2 055 481	19 658	4 536 495	43 385
Q2	2 355 297	22 338	1 706 833	16 188	4 062 130	38 526
Q3	2 310 208	21 811	1 546 013	14 596	3 856 220	36 408
Q4	2 235 779	21 254	1 390 347	13 217	3 626 126	34 472
Q5 (most affluent)	2 095 137	19 804	1 216 063	11 495	3 311 200	31 298
Overall	11 477 435	21 783	7 914 736	15 021	19 392 171	36 804

This table shows the total numbers and rates of hospital episodes split by type of hospital admission and deprivation group. All data are based on hospital episode statistics for year 2011/2012.

*Rate per 100 000 population.

IMD, index of multiple deprivation.

10.1136/jech-2016-207447.supp1 Supplementary appendix

The potential savings for the NHS if the costs associated with the age and sex-specific episode rates in the most affluent quintile in 2011/2012 were achieved in the other deprivation groups are given in table 2. The total cost associated with socioeconomic inequality in 2011/2012 was £4.8 billion, and there was a clear social gradient across the entire deprivation spectrum, with the largest cost observed in the most deprived group (£2.2 billion). Costs were broadly similar in men and women.

**Table 2:** Estimated cost of social inequality

IMD quintile	Female (£)	Male (£)	Total (£)
Q1 (most deprived)	1 127 006 663	1 065 236 932	2 192 243 595
Q2	706 629 004	671 287 893	1 377 916 897
Q3	410 841 645	405 654 922	816 496 567
Q4	198 794 943	19 012 169 9	388 916 642
Q5 (most affluent)*	−	−	−
Overall	2 443 272 255	2 332 301 446	4 775 573 701

This table shows the difference in inpatient hospital costs between those in the most affluent group and each of the other deprivation groups assuming everybody in the other groups would have the same average hospital costs as those in the most affluent groups adjusted for the different demographic profiles of the groups. All data are based on hospital episode statistics for year 2011/2012.

*Comparator group—costs in this group are £2 608 800 295, £2 208 982 887 and £4 817 783 181 for women, men and total, respectively.

IMD, index of multiple deprivation.

### Estimates of lifetime costs

Survival curves for men and women by deprivation quintile are shown in figure 2A. People who lived in more affluent areas were expected to live longer than those who lived in more deprived areas, and women were expected to live longer than men at any given deprivation level.

Combining data on survival and average costs, we calculated expected costs of hospital admission over the life course for each deprivation group, assuming survival and costs remained constant at 2011/2012 levels. Cumulative lifetime costs are shown in figure 2B. Average lifetime costs for men ranged from £43 358 for the most affluent group to £50 163 for the most deprived, and the respective costs for women ranged from £48 409 to £59 255. Overall, women had 14% higher expected lifetime hospital costs than men, largely due to the increased costs associated with the reproductive years, but also due to their longer life expectancy. Despite having longer life expectancy, people living in the most affluent fifth of areas had lower lifetime hospital costs than those living in more deprived areas.

Analyses for emergency and elective admissions are presented in the online supplementary appendix figures A1 and A2. Results were broadly similar to those for all admissions, but expected cumulative lifetime costs for elective episodes in men converged and were highest for people living in the most affluent fifth of areas. Results for outpatient appointments are also given in the online supplementary appendix figure A3. Very similar trends were apparent to those for inpatient admissions, with outpatient hospital use increasing with greater deprivation level and age, and spiking for women between the ages of 20 years and 40 years.

## Discussion

### Summary of key findings

In this study, we aimed to quantify the hospital care costs to the NHS of socioeconomic inequality. As expected, we found that hospital admission rates generally increased with age, and were higher in women during the reproductive years and higher in men at most other ages. For all ages, there was a clear socioeconomic gradient, particularly for emergency admissions, with the rate of admissions increasing with neighbourhood deprivation. The costs to the NHS associated with this inequality were partially offset by lower life expectancy in more deprived groups, but remained substantial: £4.8 billion per year at 2011/2012 levels.

### Strengths and limitations

This is the first study based on comprehensive whole-population data in England to explore the relationship between lifetime hospital costs to the NHS and socioeconomic inequality. We used data at small-area level to minimise, as far as possible, the risk of ecological fallacy that may have masked inequality at larger and coarser geographical levels. Mortality data were used to extrapolate the results of the analysis across the patient lifetime to allow conclusions to be drawn on both cross-sectional and lifetime costs of inequality to the NHS.

The study is subject to several limitations. First, we did not control for differing need for healthcare among the different groups, and so do not make any judgements on whether the different levels of healthcare use are ‘fair’ or appropriate, given differences in need. For example, it may be the case that for any given level of morbidity, poorer patients use less healthcare than richer patients, and hence, our estimate of the cost of inequality to the NHS, while representing current practice, underestimates ideal practice where patients receive equal treatment for equal need. Second, the focus of our analysis was inpatient care, but healthcare costs are also incurred through outpatient appointments and in primary care. In 2011/2012, inpatient costs and primary care costs each constituted 22% of the total NHS budget of £101.42 billion.^14^ In our supplementary analyses, we found that outpatient healthcare use followed trends similar to those for inpatient use. This suggests that our estimates represent a lower bound on the total cost of inequality to the NHS. Third, our lifetime extrapolation assumes that hospitalisation rates and costs observed in 2011/2012 will remain constant into the future, and that mortality rates in 2011/2012 can be used to predict survival rates in future years. The extrapolation also assumes that deprivation levels are fixed over individuals’ lifetimes. While these assumptions may not hold in practice we feel they give a reasonable indication of the relative magnitudes and directions of future trends. Fourth, the underlying population and mortality data breakdowns that we use in this study are truncated at 85 years of age, so mortality and hospitalisation rates for older age groups are assumed to be constant and not to increase further with age. Finally, while we use small-area-level deprivation in our analysis, to fully guard against ecological fallacy, individual-level deprivation data would be required. Such data are not available in a form that can be linked to health data in England. This remaining potential for ecological fallacy is likely to bias our estimate of the costs of inequality downwards.

### Comparison with other studies

As far as we know, this is the first published analysis of the inpatient costs of socioeconomic inequality in England. The 2010 Strategic Review of Health Inequalities (the Marmot Review) estimated the cost of inequality to the NHS to be £5.5 billion per year,^4^ but the basis for this calculation and the detailed findings were not described. ONS estimated that overall NHS spending in 2011/12 was 25.3% higher for those in the lowest income quintile compared with those in the highest (spending of £1836 and £1465 respectively).^15^ However, this is an estimate based only on variation in the age and sex make-up of respondents from neighbourhoods with different levels of deprivation. By contrast, we used comprehensive national data to calculate the actual variation in healthcare costs by area deprivation, and to model lifetime costs. Our approach found that inpatient hospital costs in 2011/2012 were 31% higher for people living in the most deprived quintile of neighbourhoods compared with people living in the least deprived quintile (average annual inpatient hospital costs per resident of £597 and £455, respectively). Forget *et al*^16^ modelled lifetime healthcare costs based on the population of Manitoba, finding costs for women were 40% higher than for men. As with our study, this gap between the sexes developed during the peak childbearing years and widened at the end of life. However, while the authors described wide variations in healthcare costs between individuals, the contribution of socioeconomic factors was not assessed. Finally, Hanratty *et al*^17^ modelled socioeconomic inequalities in public expenditure on healthcare in the last year of life in Stockholm County Council. They used individual-level income data as their socioeconomic variable and found that after controlling for age, sex, diagnosis group and healthcare utilisation there was substantially greater public expenditure on higher income patients than on lower income patients. This suggests that if we were able to adjust for need and to use individual-level deprivation data in our analysis, our estimate of the cost of inequality to the NHS would be higher.

### Policy/clinical implications

Socioeconomic inequalities in the determinants of health result in both increased morbidity and decreased life expectancy. We found that the substantially higher healthcare costs accrued by residents of deprived areas throughout their lives are only slightly offset by their lower life expectancy. Evidence suggests that even in a country with universal access to healthcare, more affluent groups benefit more,^8^ ^18^ ^19^ and healthcare is not entirely equitable. If healthcare provision were to adequately meet need, the cost disparities we describe could be even greater, although better prevention and early intervention could also result in a net reduction in the costs associated with inequality, as has been found in social and educational interventions.^20^ ^21^

Rising healthcare costs in older age are largely driven by the onset of chronic disease, and the earlier onset of these diseases in poorer populations shifts the healthcare costs to younger age groups. Better primary and secondary prevention, progressively weighted towards more deprived populations, is an obvious response, but one that has proved hard to achieve. Anticipatory interventions to tackle the onset of chronic conditions in deprived neighbourhoods can result in significant patient benefit,^22^ potentially generating net savings for the health system in any given year, as well as across the lifetimes of these patients. However, while there is scope for health professionals to do more to tackle health inequalities as providers and commissioners,^23^ ^24^ the root causes of these inequalities are socioeconomic, and the healthcare system—however, equitable—can only partially alleviate their impact.^25^ ^26^ A range of recent national social and health system programmes (eg, Health Action Zones, the Quality and the Outcomes Framework) have been associated with more equitable access to high-quality care,^26^ ^27^ and in some cases, with improvements in educational and health outcomes,^28^ ^29^ but for the most part inequalities in health outcomes have persisted—or have actually worsened.^30–32^

**What is already known on this subject**

- Poorer people tend to use more healthcare at any given age, because they are sicker, but also tend to have shorter lives.
- It is not known how these two sets of inequalities interact to produce lifetime healthcare costs for different socioeconomic groups.

**What this study adds**

- There is a social gradient in both current and lifetime hospital costs. Despite dying at a younger age, people from more deprived neighbourhoods tend to require more healthcare, and cost the National Health Service (NHS) more over their lifetimes than people from more affluent neighbourhoods.
- Socioeconomic inequality cost the NHS in England £4.8 billion in 2011/2012 as a result of excess hospital admissions.
- There is a financial as well as a moral case for tackling socioeconomic inequality: reducing socioeconomic inequalities in health would reduce the excess morbidity of the poor, resulting in longer lives. Our modelling suggests that the reduction in healthcare costs resulting from reducing morbidity among the poor would outweigh the increase in healthcare costs resulting from their increased longevity.
