# Supplementary material for: The costs of inequality: whole-population modelling study of lifetime inpatient hospital costs in the English National Health Service by level of neighbourhood deprivation
Source: J Epidemiol Community Health. 2016 May 17;70(10):990–6. doi: 10.1136/jech-2016-207447 (PMC5036206; doi:10.1136/jech-2016-207447)
Supplement: Supplementary appendix [file jech-2016-207447supp_appendix.pdf]

The costs of inequality: whole-population modelling study of lifetime inpatient hospital costs in the English National Health Service by level of neighbourhood deprivation

Supplementary Appendix

This supplementary appendix provides additional details about the social gradient in hospital utilisation and associated costs in terms of type of inpatient appointments (elective versus emergency) as described in **Figures A1** and **A2** as well as describing the social gradient in use of outpatient care as described in **Figure A3**.

Figure A1: Hospital inpatient admissions split by admission type, age, sex and deprivation

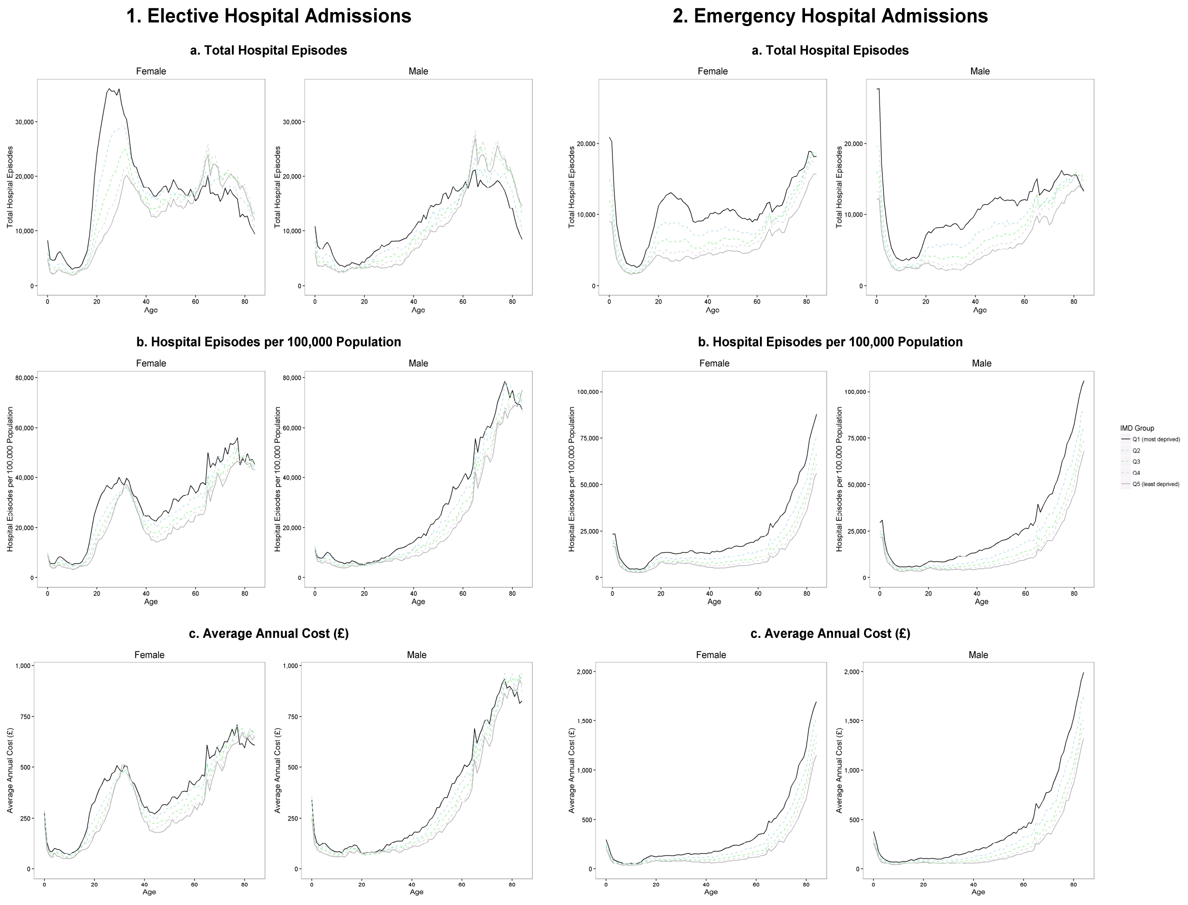

Notes to figure A1: Graphs are based on hospital episode statistics for year 2011/12 and are broken down by admission type (set of graphs on the left show elective admission while those on the right show emergency admissions), sex (female on the left within each admission type and male on the right), deprivation (different line colours) and are plotted against age. **Panel a** shows the total number of hospital episodes. **Panel b** shows the hospitalisation rate i.e. adjusts for the demographic structure of the population. **Panel c** translates from hospital episodes to average annual costs due to these hospitalisation.

Figure A2: Hospital inpatient costs broken down by admission type age, sex and deprivation

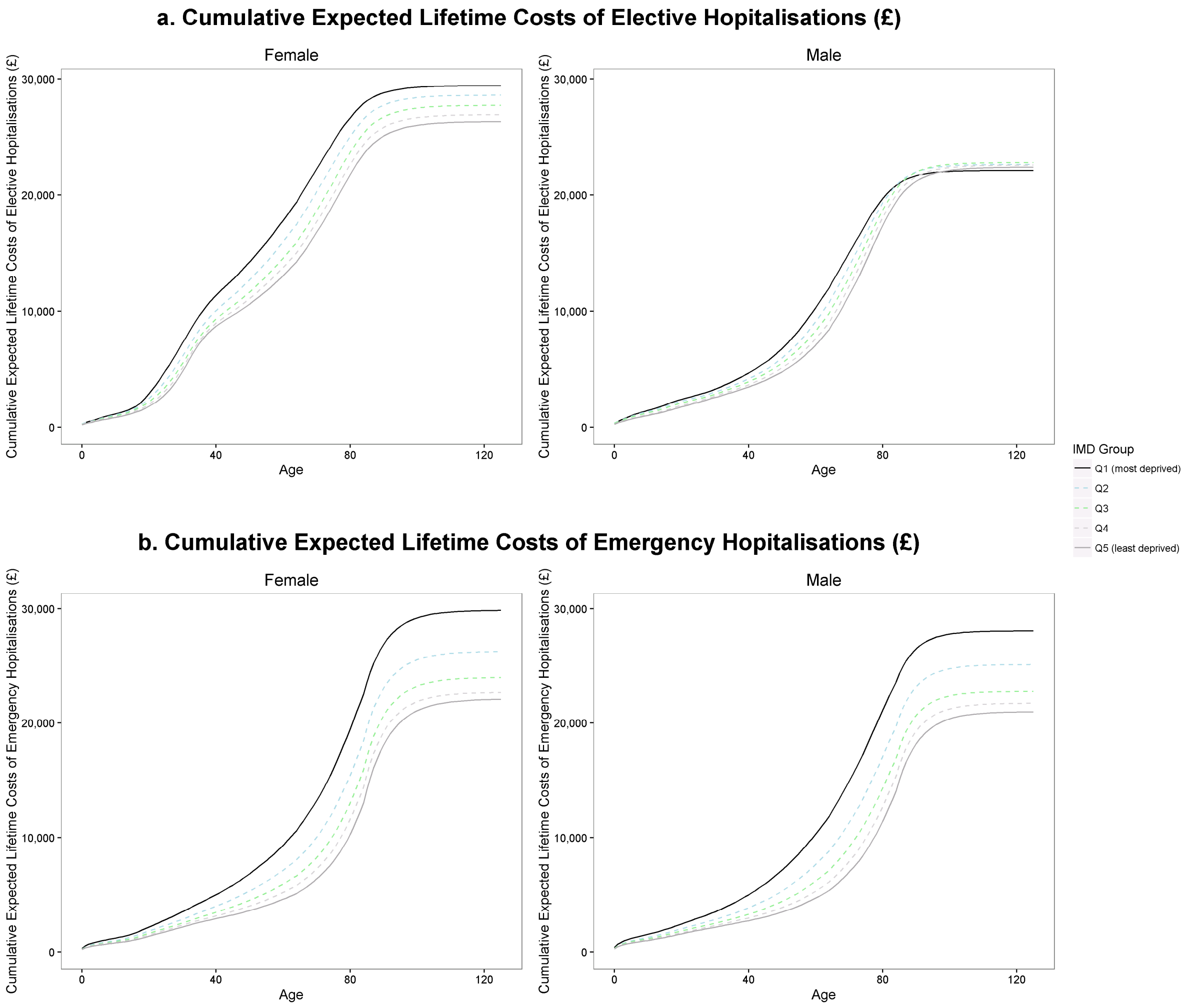

Notes to figure A2: Graphs are based on and mortality data and hospital episode statistics for year 2011/12 and are broken down by sex (female on the left male on the right), deprivation (different line colours) and are plotted against age. **Panel a** shows cumulative expected hospital costs due to elective hospital episode and **Panel b** shows the cumulative expected hospital costs due to emergency hospital episodes. These costs are calculated by adjusting hospital costs by survival probabilities and cumulating these adjusted costs over all previous years.

Figure A3: Outpatient appointments split by age, sex and deprivation

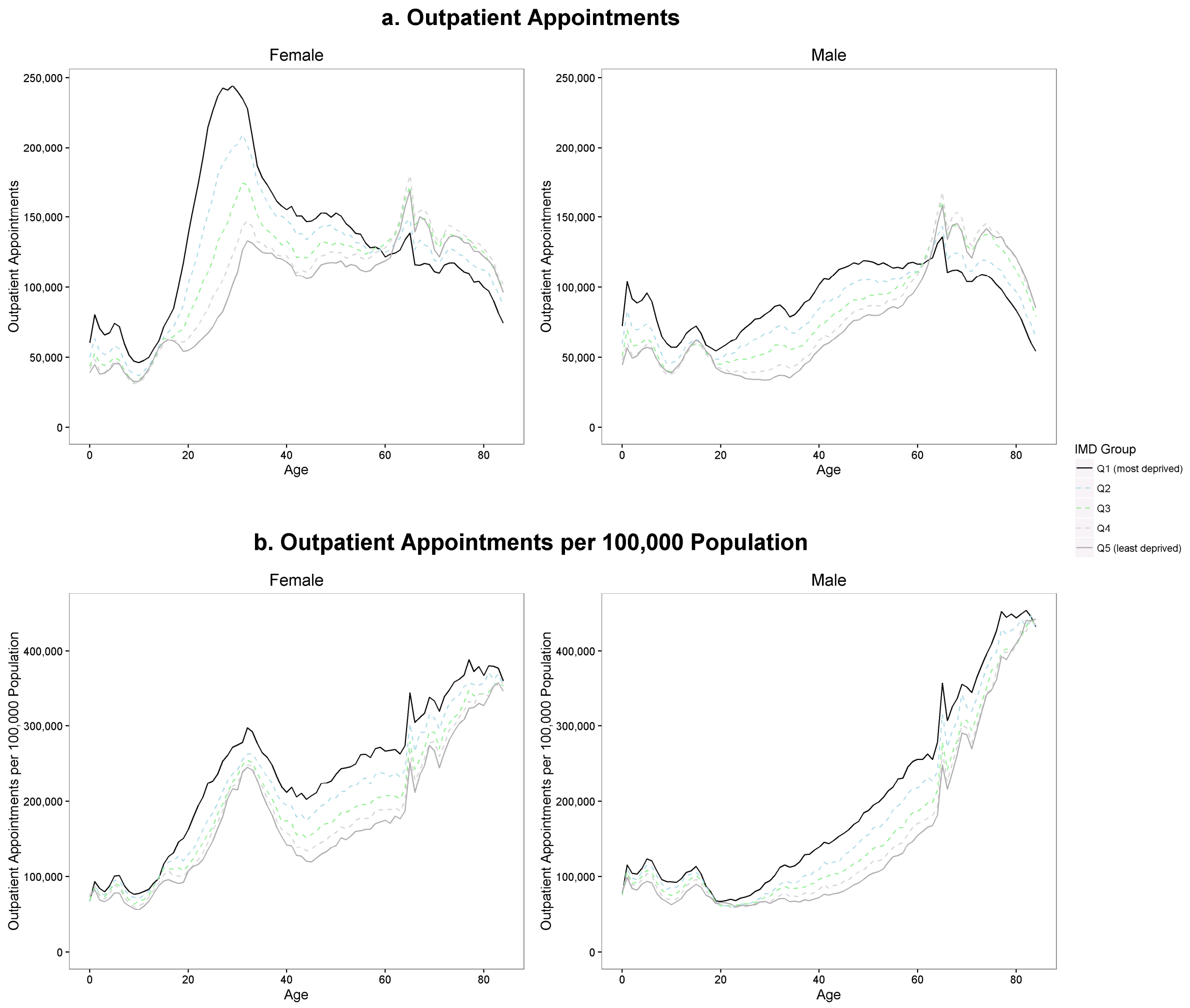

Notes to figure A3: Graphs are based on hospital episode statistics for year 2011/12 and are broken down by sex (female on the left male on the right), deprivation (different line colours) and are plotted against age. **Panel a** shows the total number of outpatient appointments. **Panel b** shows the appointment rate i.e. adjusts for the demographic structure of the population.
